# Nuclear-specific gene expression in heterokaryons of the filamentous ascomycete *Neurospora tetrasperma*

**DOI:** 10.1098/rspb.2022.0971

**Published:** 2022-08-10

**Authors:** Cécile Meunier, Iulia Darolti, Johan Reimegård, Judith E. Mank, Hanna Johannesson

**Affiliations:** ^1^ Department ECOBIO, UMR CNRS 6553, Université Rennes 1, Rennes, France; ^2^ Department of Zoology and Biodiversity Research Centre, University of British Columbia, Vancouver, Canada; ^3^ Department of Cell and Molecular Biology, National Bioinformatics Infrastructure Sweden, Science for Life Laboratory, Uppsala University, Uppsala, Sweden; ^4^ Department of Organismal Biology, Uppsala University, Uppsala, Sweden; ^5^ Centre for Ecology and Conservation, University of Exeter, Penryn Campus, UK; ^6^ The Royal Swedish Academy of Sciences and Department of Ecology, Environment and Plant Sciences, Stockholm University, SE-106 91 Stockholm, Sweden

**Keywords:** heterokaryosis, additivity, nuclear interaction, allele-specific expression

## Abstract

Heterokaryosis is a system in which genetically distinct nuclei coexist within the same cytoplasm. While heterokaryosis dominates the life cycle of many fungal species, the transcriptomic changes associated with the transition from homokaryosis to heterokaryosis is not well understood. Here, we analyse gene expression profiles of homokaryons and heterokaryons from three phylogenetically and reproductively isolated lineages of the filamentous ascomycete *Neurospora tetrasperma*. We show that heterokaryons are transcriptionally distinct from homokaryons in the sexual stage of development, but not in the vegetative stage, suggesting that the phenotypic switch to fertility in heterokaryons is associated with major changes in gene expression. Heterokaryon expression is predominantly defined by additive effects of its two nuclear components. Furthermore, allele-specific expression analysis of heterokaryons with varying nuclear ratios show patterns of expression ratios strongly dependent on nuclear ratios in the vegetative stage. By contrast, in the sexual stage, strong deviations of expression ratios indicate a co-regulation of nuclear gene expression in all three lineages. Taken together, our results show two levels of expression control: additive effects suggest a nuclear level of expression, whereas co-regulation of gene expression indicate a heterokaryon level of control.

## 1. Introduction

Genetic variation within organisms has been shown to occur at all major branches of multicellular life [1–3]. These findings challenge the concept of the individual as a single unit of selection [2], and arguments have been raised that within-organism variation benefits the organism, either by providing phenotypic flexibility to a changing environment [3,4] or by allowing purging of deleterious cell lineages [5]. In the fungal kingdom, within-organism variation in the form of heterokaryosis dominates the life cycle of many species. Heterokaryosis is a genetic system in which genetically distinct nuclei coexist within the same cytoplasm. Heterokaryosis can result from mutations in one or more nuclei in a homokaryotic mycelium, or from fusion between hyphae of genetically distinct mycelia. The life cycle of most basidiomycetes and certain ascomycetes includes a long-lived heterokaryotic mycelium [6,7] that originates through fusion (mating) of homokaryotic individuals which carry nuclei of compatible mating types [8]. In the heterokaryotic cell type resulting from mating, nuclei remain haploid and separated, which contrasts with the predominant outcome of sexual reproduction in plants and animals where karyogamy immediately follows plasmogamy [9,10].

Recent and ongoing work has revealed two fundamental challenges associated with the heterokaryotic life history of filamentous fungi: the coordination of populations of nuclei for growth and development, and the suppression of nuclear competition during reproduction and dispersal [11–14]. In many basidiomycetes, a 1 : 1 nuclear ratio in the heterokaryon that results from mating is usually controlled by clamp connections [6] making it strictly dikaryotic and equivalent to diploidy in having an equal number of genotypes in cells and tissues. However, nuclear dominance in both gene expression and relative abundance has been documented in heterokaryons. For example, dominance in gene expression has recently been confirmed in heterokaryons of the basidiomycete *Agaricus bisporus* [15], and a biased nuclear ratio has been reported both in the basidiomycete *Heterobasidion parviporum* and in the ascomycete *Neurospora tetrasperma* [13,14,16]. Furthermore, nuclei of many species of filamentous fungi are capable of dividing and moving independently and freely through septal pores to traverse the interconnected syncytium, with a rate that can reach several microns per second [12], and they are able to spread on their own via mycelial interactions and/or unicellular asexual spores and conidia [17]. These findings further point toward the room for independent evolution of the nuclei of a heterokaryon.

Despite the prevalence of heterokaryosis among fungi, interactions among the distinct nuclei within the heterokaryon have remained largely uncharacterized. The transition from homokaryosis to heterokaryosis for mating type involves the obvious changeover from sterile to fertile tissue. However, phenotypic and gene expression differences are also often identified at the vegetative stage. For example, mycelial cultures of homokaryons have been reported to differ from heterokaryons in both morphological appearance and growth rate (e.g. [13,18]), suggesting that a developmental switch is mediated by the interaction of the two genomes when they coexist in the heterokaryon. Genome-wide expression differences between homo- and heterokaryons have also been identified in, for example, the basidiomycete *Pleurotus ostreatus* [19]. Such a process is analogous to hybrids in which mating of two individuals of different species results in novel gene interactions that lead to transgressive gene expression as compared to parents [20–22], suggesting heterokaryon-level control of phenotypes and expression. On the other hand, previous studies have shown that the heterokaryon phenotype is additively associated with the nuclear ratio [14]. These observations are expected if nuclei express genes independently with no or minimal heterokaryon-level of control. Furthermore, we have previously found evidence that the two heterokaryon nuclei have complementary traits, consistent with division of labour and cooperation to optimize overall fitness [14]. These latter observations support the idea that heterokaryon phenotypes reflect the underlying nuclear composition, and that the heterokaryon can adapt to a changing environment merely by changing nuclear ratios in a manner that reflects the underlying relative fitness of the constituent homokaryons grown in isolation. Heterokaryosis then becomes an advantage *per se*.

In this study, we analyse the molecular phenotype, i.e. the transcriptome, of homo- and heterokaryons of the filamentous ascomycete *Neurospora tetrasperma* grown at different stages of the life cycle. *N. tetrasperma* has evolved a novel genetic system in which heterokaryosis is associated with mating types, and hence, haploid nuclei of opposite mating types, *mat A* and *mat a*, coexist within cells throughout the life cycle. While the nuclei of the two mating-types in a natural heterokaryon show very low divergence between a majority of the chromosomes, suppression of recombination around the mating-type (*mat*) locus results in linkage of over 1500 genes, and thus, to the nuclear type [23–25]. Accordingly, sequence divergence between the linked region of *mat A* and *mat a* is as high as 3.2% [23–25] as a result of both mutation accumulation and, in certain lineages, introgression from closely related species [25,26]. *N. tetrasperma* consists of multiple, reproductively isolated lineages, and mating-type nuclei have diverged numerous times independently. Previous studies have indicated that *mat A* and *mat a* nuclei of *N. tetrasperma* heterokaryons are distinctly specialized. First, although they are packaged together into the sexual spores in a 1 : 1 proportion [7], the germinating mycelium often shows a non-random pattern of biased nuclear ratio [13,14]. Second, phenotypic differentiation has been documented for the two nuclear types, and trade-offs between fitness characteristics is consistent with division of labour and cooperation [14]. Hence, as *N. tetrasperma* typically grows as a heterokaryon and is able to go through the life cycle without outcrossing [27], it is possible that the two nuclei of a strain have co-evolved and adapted to each other over long periods of time. This presents the opportunity for a dynamic that differs from homokaryotic species, in which nuclei encounter each other and cohabitate on a more transient basis [14]. Of particular note, phenotypic differentiation linked to mating type varies among reproductively isolated lineages, suggesting coevolution has taken place several times independently. Finally, gene expression differences between the two nuclei of *N. tetrasperma* have also been found primarily on the mating-type chromosome [28], and expression analysis of six genes linked to mating-type suggests that expression is coregulated between the nuclei in the heterokaryon to obtain a tissue-specific bias in expression ratio [13].

Using *N. tetrasperma* as a model system, we setup to explore whether patterns of expression reflect a heterokaryon-level of control, or relate more to independent expression of nuclear types. To this aim, we collected both vegetative and sexual tissue from three phylogenetic lineages of *N. tetrasperma,* to test if the level of expression control differs between developmental stages*.* We measured differential global gene expression between homokaryons and heterokaryons, and allele-specific expression within the heterokaryon to be able to infer coregulation between nuclei within the heterokaryon. With this data, we asked three broad questions. First, we investigated whether gene expression in *N. tetrasperma* is primarily determined by developmental stage, lineage, or nuclear composition. Because heterokaryosis allows completing the life cycle of each individual, we expected a heterokaryon-level of control of expression to translate in differential expression mostly driven by developmental stage, rather than by nuclear type. Second, we asked whether heterokaryon gene expression shows a developmental switch relative to homokaryons, or if genes are expressed in an additive manner. Finally, we assessed whether genes of the two nuclei in the heterokaryon are coregulated or expressed independently, and if this depends on developmental stage. This last analysis can reveal if nuclear ratios passively drive expression ratios or whether expression ratios differ from nuclear ratios, suggesting heterokaryon-level coregulation between nuclear types.

## 2. Methods

### (a) Fungal material used in the study

We used six haploid and homokaryotic strains of *Neurospora* that belong to three phylogenetically and reproductively isolated lineages of *N. tetrasperma* (L1, L6 and L10). The pairs of homokaryons were isolated in previous studies from naturally occurring heterokaryons as indicated in electronic supplementary material, table S1. All strains are available at the Fungal Genetics Stock Center (FGSC), University of Missouri, Kansas City.

### (b) Preparation of inocula to create tissues of *N. tetrasperma* with different nuclear composition

Asexual spores, conidia, of the fungus fuse after germination and were used in this study to generate the inoculum for the investigated tissue of *N. tetrasperma*. For homokaryotic samples, we used single-mating type conidia yielding either *mat A* and *mat a* homokaryotic tissue. Conidia of compatible genotypes were used to generate heterokaryons of *N. tetrasperma* with predetermined ratios of nuclei of the two mating types. Following the method outlined in Meunier *et al*. [14], we used conidial mixes to generate heterokaryotic inocula with three different initial nuclear contents: 90% *mat A*, 50% *mat A* and 10% *mat A*. We verified that the conidial mixes resulted in heterokaryotic mycelia by isolating hyphal tips and verifying self fertility of the developing mycelium [14]. As nuclear ratios of heterokaryons may deviate from the inoculum over growth, we ascertained the observed nuclear ratio over the experiment by using qPCR estimations. Specifically, we used primers designed for a clear discrimination between the alleles linked to *mat A* or *mat a*. After qPCR amplifications, built-in methods allow calculating DNA starting quantities of *mat A* and *mat a* in the samples, from which we deduced mating-type ratios (see detailed protocol in [14]).

### (c) Growth conditions promoting different developmental stages

**Figure 1.**
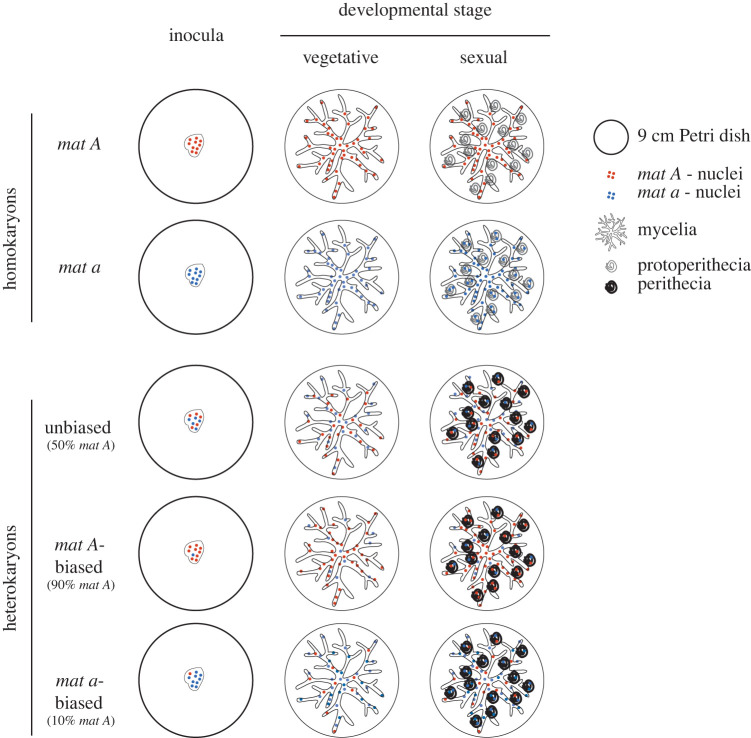
Experimental design of the study. From each of the three *N. tetrasperma* lineages, we investigated tissues from two developmental stages: vegetative (grown on Vogel's Medium N over two days) and sexual development (grown on modified Vogel's Medium N over seven days), as illustrated schematically. Homokaryotic tissues include only one or the other nuclear type (*mat A* or *mat a*), while heterokaryotic tissues, which contain both nuclear types within the same cells, were generated by constructing inoculates with different nuclear ratios (90%, 50% or 10% *mat A*). For both homokaryons and heterokaryons, tissues from the vegetative stage consist of elongated hyphae connected into mycelial networks. These mycelia bud off the asexual spores, conidia (not shown on the drawing). As homokaryons contain only one mating type, they are sterile, and at the sexual developmental stage they produce immature fruiting bodies, protoperithecia, as structures in the mycelium. The heterokaryons on the other hand, are fertile, and during the sexual stage protoperithecia mature into fertile fruiting bodies, perithecia, in which nuclear fusion occurs between opposite mating type nuclei and meiosis can take place. Note that the tissues from both the vegetative and the sexual developmental stages contain mycelia and conidia, and hence, they are not completely different. For each lineage, three biological replicates were included for each nuclear composition and tissue type, resulting in a total of 30 samples for which RNA was extracted, sequenced and analysed. (Online version in colour.)

The experimental design is shown in figure 1. The centers of 90 mm Petri dishes were inoculated with 20 µl of conidia suspensions and emerging mycelia were grown at 25°C, with a 12 : 12 light-dark cycle. We investigated tissues grown under two different conditions; (i) on Vogel's Medium N [29] over 2 days, promoting vegetative growth (the condition hereafter referred to as the ‘vegetative stage’) and (ii) on modified Vogel's Medium N [30], over seven days, promoting ‘sexual development’ (sexual developmental stage). For all media, we used sucrose (1%) as a carbon source and agar (1.5%) for solidification, and the agar surface was covered with cellophane to facilitate harvesting. At the end of each treatment, tissue was harvested from the surface of the Petri dishes using a sterilized scalpel. Tissue from the same plate was divided into two parts: one part of the tissue was stored at −20°C for DNA extraction and qPCR, and the second part immediately frozen in liquid nitrogen and stored at −80°C for RNA extraction. As biological replicates, we used tissue grown on independent Petri dishes using the same source inoculum and growth condition. We incorporated three biological replicates in the design, resulting in a total of 30 samples of each lineage for which RNA data was gathered and analysed.

### (d) Genome resources and identification of orthologous genes

For each strain used in this study, we downloaded the genome sequence and assembly from relevant genome resources [24,31,32] (electronic supplementary material, table S1). Genomes from L1 (*mat A* and *mat a*), L10 (*mat A* and *mat a*) and L6 (*mat A*) were all aligned to the *N. tetrasperma* L6 *mat a* genome using MUMmer (v. 3.9.4alpha) [33]. We used the annotations presented by Hosseini *et al*. [34], and OrthoMCL [35] to generate orthologue clusters among the genomes of strains listed in electronic supplementary material, table S1. We chose the granulometry yielding the maximum number of orthologues shared by all strains, and retrieved 7099 orthologous genes.

### (e) Determination of global expression level

For each biological replicate, total RNA was extracted and sequenced using the protocol outlined in [34]. RNA reads were filtered for rRNA content, trimmed using Trimmomatic v. 0.32 [36] and mapped to a genome of their respective lineage using STAR 2.5.1b [37], resulting in average mapping efficiency of 60–70%. Read counts were extracted using featureCounts from the subread package [38] and levels of expression were analysed using the package edgeR in R [39] following removal of lowly expressed genes and normalization of libraries sizes. For all analyses requiring a comparison between strains, after building the DGEList, counts within coding sequences were normalized across libraries of all strains using orthologues. The obtained normalization factors were then used on each library strain.

### (f) Clustering and PCA analyses

We used clustering and PCA analysis to infer the similarity of expression among tissue types and lineages. We used the package DESeq2 from Bioconductor v. 3.11 in R [40], which provides methods to test for differential expression by use of negative binomial generalized linear models. Counts were transformed on a log scale accounting for normalization factors for visualization and clustering purposes. We used the rlog method in DESeq2, which is more robust when size factors vary widely. The ‘Blind’ argument (to the sample information in the design) was set to ‘False’ because the goal was not sample quality insurance, but assessment of expression differences among treatments. Clustering analysis was then performed using a Euclidean method to compute distances among transcriptomes and PCA plotted using a custom script.

### (g) Analyses of homokaryon versus heterokaryon gene expression in vegetative tissue

The R package edgeR [39] was used to estimate differential expression between homokaryons, and between homokaryons and the heterokaryons with three different nuclear ratios (table 1). Briefly, the function fits a quasi-likelihood negative binomial generalized log-linear model to read count data for gene-by-gene statistical tests of differential expression. Focusing on the data obtained from the vegetative stage, we determined the number of genes with conserved expression as opposed to genes differentially expressed in at least one treatment. We next estimated the number of genes within the latter group that showed differential expression between homokaryons of different mating type. These genes were further classified into three categories. The first category includes those genes showing additive expression, i.e. genes showing expression consistent with the nuclear ratio in the heterokaryon. Second, we categorized genes as dominant when showing expression significantly different from one homokaryon but not different from the other, and significantly different from additivity of nuclear ratio. Third, transgressive genes, i.e. over- or under-dominant, refers to the category of genes showing significantly higher or lower expression than both homokaryons of the respective lineage.

**Table 1.** Number and proportion of genes with different modes of expression in heterokaryons as compared to homokaryons of the vegetative stage of development. The total amount of expression (i.e. *mat A* + *mat a*) of each gene in heterokaryons is compared to the amount in each homokaryon. Percentages are given as proportion out of the number of genes in the group that is one level higher.

	L1	L6	L10
total number of genes expressed	7846	7800	7767
genes with conserved expression	4596 (58.6%)	7534 (96.6%)	1899 (24.4%)
genes without conserved expression	3250 (41.4%)	266 (3.4%)	5868 (75.6%)
genes differing in expression between *mat A* and *mat a*	2114 (65.0%)	244 (91.7%)	4550 (77.5%)
additive expression^a^	2102 (99.4%)	229 (93.8%)	2273 (50.0%)
non-additive expression^b^	12 (0.6%)	15 (6.1%)	2277 (50.0%)
genes with another pattern of expression difference	1136 (35%)	22 (8.3%)	1318 (22.5%)

### (h) Identification of genes with biased expression

In each lineage, we used edgeR in R [39] to analyse gene expression data of the homokaryons only and extract genes significantly higher expressed in one mating-type homokaryon versus the other (defining *mat a*-biased and *mat A*-biased genes, respectively) and genes significantly higher expressed in one stage of development compared to the other (vegetative-biased and sexual-biased genes). Genes were selected based on a log_2_ fold change greater than 1 and a *p* value < 0.05. We used chi-squared tests in R to determine if the overlap between *mat*-biased and stage-biased genes was greater than expected by chance in any of the lineages.

### (i) Nucleus-specific RNA mapping for expression ratios within the heterokaryons

The expression ratio of genes of the two nuclei within each heterokaryon was computed based on RNA reads. RNA reads were mapped using STAR v. 2.5.1b to a combined *mat A* and *mat a* genome for each heterokaryon, then orthologues counts were obtained using featureCounts from the subread package. For those genes which are identical in sequence between both heterokaryons, there were no uniquely mapped reads. For these we were unable to infer nucleus-specific expression. We filtered out orthologues for which mismapping was observed (filtering threshold of 5% mismapping), using reads generated in homokaryons to estimate the accuracy of mappings.

### (j) Analysis of interaction between nuclear ratio and expression ratio

Across heterokaryotic tissues of every lineage and developmental stage, we investigated the interaction between the relative abundance of the mating type nuclei (i.e. *mat A*/*mat a* nuclear ratio) and the relative expression of alleles of the two nuclei (i.e. *mat A*/*mat a* RNA expression ratio) by correlating the proportion of *mat A* in the DNA against that in the RNA. Specifically, we linearly modelled the slope and intercept for each gene individually using lm in R stats package that produces the most probable slope and intercept value. It also produces a confidence interval for the slope and the intercept to address the uncertainty of the model. We identified genes in the homokaryons data where the confidence interval of the slope did not overlap with 1, i.e. for which there were not a one to one correlation between DNA and RNA concentrations,. These were assumed to represent genes with mapping issues to the combined genome assembly and were removed from further analysis. Second, we identified the genes with biased expression within the heterokaryon. Here, we used the heterokaryons data to identify genes where the interval of the slope did not overlap with 1, i.e. that did not show a one to one correlation between DNA and RNA concentrations. On a global level we did the Mann-Whitney test, using wilcox.test in R stats package, on the modelled slope values to identify differences in distributions. More specifically, for each lineage we compared the slope distributions, using heterokaryons data, of the vegetative to the sexual tissue. To address significant changes at the gene level dependent on the developmental stages we also tested for differences in slope and intercept between the two. Using the replicates data for each gene in the sexual stage, we calculated the slope Z-score as (slope_gene_ − mean slope_sexual stage_)/slope standard deviation_vegetative stage_. Similarly, the intercept Z-score for each gene was calculated as (intercept_gene_ − mean intercept_sexual stage_)/intercept standard deviation_vegetative stage_. Z-score values greater than 2 and values <−2 indicate a significantly greater and, respectively, lower slope or intercept in the sexual stage compared to the vegetative stage.

## 3. Results

### (a) *N.* *tetrasperma* gene expression is primarily determined by developmental stage

**Figure 2.**
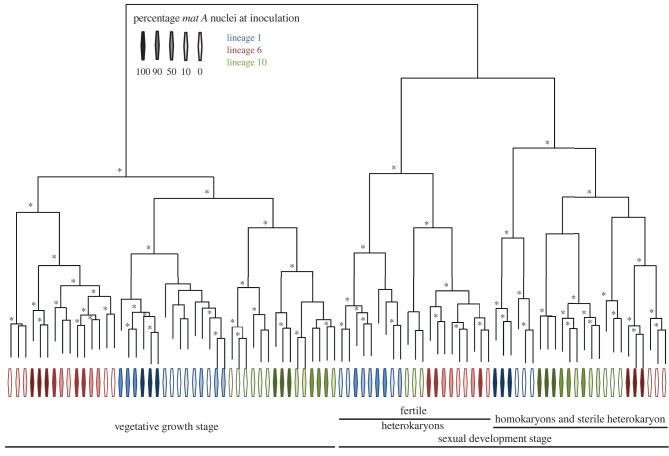
Hierarchical clustering of gene expression for homokaryons and heterokaryons with different nuclear ratios of the three *N. tetrasperma* lineages. Each lineage is represented through a different colour, with darker colour shadings indicating a higher *mat A* nuclear proportion. Hierarchical clustering is based on Euclidean distance for expression for each orthologous gene. Nodes with greater than 95% bootstrap support are marked with an asterisk. (Online version in colour.)

Hierarchical clustering of gene expression profiles revealed that developmental stage is the most important determinant of gene expression. First, independent of lineage and nuclear content, gene expression profiles form two main clusters, separating samples grown on media promoting vegetative versus sexual development (figure 2). Furthermore, within sexual samples, expression differs between sterile or fertile tissues. Sterile tissue includes sexually maturing homokaryons with nuclei of only one mating type, while fertile tissue includes heterokaryons harbouring both *mat a* and *mat A* nuclei (figure 1). An exception to this are the heterokaryons of L10 with a strong final *mat A* nuclear bias, which have been previously shown to be largely sterile in spite of both mating types being present in the tissue [14]. Within each developmental stage and tissue type, expression further clusters by lineage (figure 2). Within the cluster of samples from the vegetative growth stage, the strains group in accordance with previous findings on relatedness, with L1 and L10 clustering more closely to each other than each of them with L6. By contrast, L6 and L10 show most similar expression profiles at the sexual stage, a pattern that is observed both within fertile heterokaryons, homokaryons and sterile heterokaryons (figure 2).

PCA plots confirm the pattern seen with the cluster analysis (electronic supplementary material, figure S1–S3), in that developmental stage explains most of the variance in gene expression. When plotting PC1 against PC2 for L1 and L6, respectively, we see three clusters, again separating (i) vegetative, (ii) sexually maturing but sterile and (iii) sexually maturing fertile heterokaryons (electronic supplementary material, figure S1A and B). For L10, the sterile heterokaryons with 50–90% *mat A*-nuclei cluster in yet another group (electronic supplementary material, figure S1C). It is noteworthy that the PCAs also show variance in expression for nuclei of the two mating types, mostly during the sexual stage, as homokaryons (100% *mat A* and 100% *mat a*) cluster separately, and heterokaryons cluster by their initial ratios. This mating-type ratio effect is, however, mostly seen on PC3, which explains only a small percent of the variance (electronic supplementary material, figure S2). Lineages also cluster apart in all-lineage PCAs (electronic supplementary material, figure S3), for which we plotted the data from the vegetative and sexual developmental stages separately.

### (b) Gene expression in heterokaryons shows additivity

In order to investigate whether a developmental switch is mediated by the interaction of the two genomes within the heterokaryon, we compared gene expression between homokaryons and heterokaryons of each lineage. In these analyses, we compared total expression for each gene rather than allele-specific patterns. We also restricted these specific analyses to those genes expressed at the vegetative stage, as the developmental difference between heterokaryons and homokaryons under sexual maturation (producing fertile versus sterile tissue, respectively; figure 1) leads to observed differences in expression due to factors other than nuclear organization.

For each lineage, we first determined the number and percentage of expressed genes with conserved expression, i.e. that did not differ among homokaryons and heterokaryons with different nuclear ratios. We then focused subsequent analyses on the non-conserved genes which showed differential expression between homokaryons of different mating type to infer whether the expression in the heterokaryon was additive, dominant, or transgressive (i.e. over- or under-dominant) relative to the homokaryons. Given that heterokaryons do not show a 50 : 50 ratio of the homokaryotic genomes, we took nuclear ratio into account when contrasting expression in heterokaryons to expression in both homokaryons. Our results revealed that expression patterns in heterokaryons relative to homokaryons differ between lineages. In L6, the vast majority of the genes (96.6%) show conserved expression between homokaryons and heterokaryons. The great majority of remaining genes show expression patterns consistent with additive expression consistent with nuclear ratio (table 1). In L1, although more genes depart from conserved expression (41.4%), and a higher number of genes show differential expression between mating types, the vast majority of the latter are also consistent with additive expression (table 1). By contrast, L10 exhibits a great number of genes that depart from conservation (75%), with approximately 77% of these showing differential expression between mating types. Of these latter genes, only 50% show strict additivity, while other genes show intermediate patterns, with few (36) fitting the pattern of dominance or transgressivity (table 1; electronic supplementary material, table S2). Hence, even if the number of genes showing dominance or transgressivity in L10 is low, it is different from the other lineages in that gene expression in the heterokaryon is not merely a reflection of the relative amount of nuclei of the two mating types (electronic supplementary material, table S2).

### (c) Patterns of coregulation of genes between nuclei

**Figure 3.**
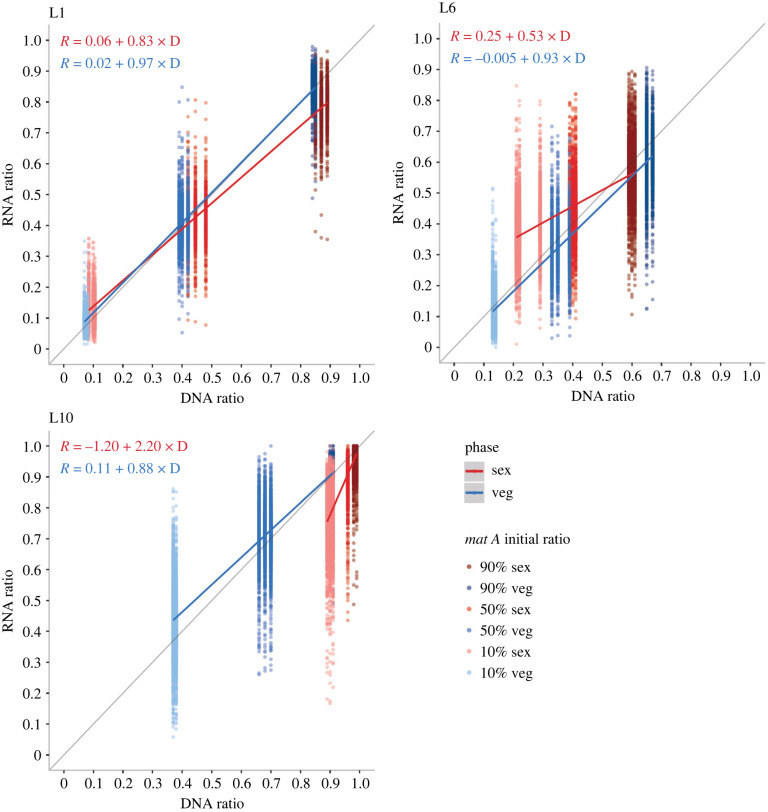
Regression between the proportion of nuclei in the heterokaryotic mycelia (DNA ratio) and the expression ratio of the two nuclei (RNA ratio) for sexual (red) and vegetative (blue) stages of development. Darker colour shadings of data points indicate a higher *mat A* initial nuclear ratio. For each lineage, the linear regression line and equation for the sexual and vegetative datasets are shown in red and blue, respectively, where R represents the RNA ratio and D the DNA ratio. The diagonal grey line represents a line with slope 1 and intercept 0.

For both vegetative and sexual heterokaryotic tissue of every lineage, we called nucleus-specific DNA and RNA content, and used this information to examine the interaction between *mat A*/*mat a* DNA versus RNA ratio for each gene individually. In all three lineages, the nuclear ratio correlates with the expression ratio in the vegetative stage, with an average slope across all genes close to 1 and an average intercept close to 0 (figure 3; electronic supplementary material, figure S4, table S3). Indeed, in the vegetative stage, the vast majority of genes in both L1 and L6, and half of the genes in L10, have a slope confidence interval overlapping 0 (electronic supplementary material, table S4), suggesting that at this stage of development *mat A* and *mat a* nuclei are expressed independently of each other, and expression ratios strongly depend on nuclear ratio.

By contrast, in the sexual developmental stage the average slope of the regression analysis is significantly different from 1 in all three lineages (figure 3; electronic supplementary material, figure S4, table S3), suggesting a strong pattern of coregulation of genes between nuclei. In L1 and L6, the majority of genes in the sexual stage exhibit a slope significantly less than 1 (electronic supplementary material, table S5, figure S4). When comparing between the vegetative and sexual stages, the distribution of slopes is significantly lower in the sexual stage (Mann-Whitney tests, *p* < 1 × 10^−16^). This difference is also reflected at the gene level (electronic supplementary material, table S6). Taken together, our data shows that in sexual development of L1 and L6, RNA is more evenly expressed among the two nuclei than expected if the genes are regulated independently of each other and of nuclear ratio. Contrary to patterns in L1 and L6, the vast majority of genes in the sexual stage of development of L10 have the slope of the regression between nuclear and expression ratios significantly greater than 1 (electronic supplementary material, table S5, figure S4). When comparing between the vegetative and sexual stages, the distribution of slopes is significantly higher in the sexual stage (Mann-Whitney tests, *p* < 1 × 10^−16^). This difference is also reflected at the gene level (electronic supplementary material, table S6). Hence, in L10, we see that the expression of genes of *mat a* is higher than expected based on additivity.

When analysing the genes located in different regions relative to the *mat* locus, we found that genes in L1 and L6 with significant slope deviations were almost exclusively found on the non-recombining region of the mating-type chromosome, while more than half of the L10 genes with significant slope deviations in the sexual stage are located on other chromosomes than the mating-type chromosome (electronic supplementary material, table S5).

Of the genes in the sexual stage without significant slope deviation, we found a large number with an intercept significantly greater than 0 in both L1 (25%) and L6 (63%) (electronic supplementary material, table S7). This indicates that for these genes, regulation of nuclei in the sexual stage takes place in order to obtain a stronger *mat A* bias in expression. In L10 however, we found only 13% of genes showing a significant departure of the intercept from 0 when in the sexual stage (electronic supplementary material, table S7).

### (d) Relationship of *mat*-biased expression and stage of development

We investigated the relationship between *mat*-biased expression and stage of development. Within each lineage, we tested the overlap between genes exhibiting an expression biased towards one mating-type (*mat a*-biased and *mat A*-biased genes) and genes with a bias in expression for one stage of development (vegetative-biased and sexual-biased genes). In L10 only, we find that compared to vegetative-biased genes, loci with a higher expression in the sexual stage of development are significantly more enriched for *mat a*-biased than *mat A*-biased genes (table 2). We also find twice as many *mat*-biased genes in L10 than we do in L1 and L6 (electronic supplementary material, table S8). These findings are consistent with previous work showing that a higher sexual fitness in L10 is associated with a *mat a*-biased nuclear ratio [14], as well as with the strong *mat a*-biased expression in the L10 sexual heterokaryon (figure 3).

**Table 2.** Differences between *mat a-* and *mat A*-biased genes among vegetative- and sexual-biased genes. Significant differences are based on Chi-squared tests in R and are shown in bold.

lineage	vegetative-biased genes		sexual-biased genes		*p*-value
	*mat a*-biased genes	*mat A*-biased genes	*mat a*-biased genes	*mat A*-biased genes	
L1	8	7	45	16	*p* = 0.219
L6	15	19	31	34	*p* = 0.899
L10	22	28	103	44	***p* = 0.002**

## 4. Discussion

### (a) Gene expression changes during the transition from homokaryosis to heterokaryosis

Like other heterokaryotic fungal species resulting from the fusion of two homokaryons carrying nuclei of different mating type, heterokaryosis in *N. tetrasperma* results in self-fertility. As such, when grown under conditions promoting sexual development, heterokaryons are phenotypically distinct from homokaryons (figure 1). In this study, we investigated whether the observed phenotypic pattern translates into differences in gene expression. In addition, as the transition from homokaryosis to heterokaryosis results in the opportunity for novel interactions between the allelic variants that coexist in the same cytoplasm, we tested whether such novel interactions result in phenotypic differences from the homokaryotic average, analogous to patterns found in hybrids versus parents [22,41,42].

First, our hierarchical clustering of homokaryons and heterokaryons gene expression revealed that tissues from the sexual stage of development do indeed show a transcriptional profile distinct from that of sterile tissue, and that conserved pattern predates diversification of the *N. tetrasperma* lineages (figure 2). These results suggest that the observed phenotypic switch to fertility in heterokaryons in the sexual stage is also reflected in changes in gene expression. The fact that sterile heterokaryon gene expression patterns cluster with the homokaryons shows that fertility versus sterility during sexual development is more important for predicting gene expression than nuclear organization. This interpretation is also supported by the finding that heterokaryons and homokaryons in the vegetative stage show no clear transcriptional separation in our clustering analysis (figure 2). Hence, our results do not support any transcriptional difference between homokaryons and heterokaryons *per se*, similar to another study in Basidiomycetes showing overall additivity but for one gene [43].

Furthermore, our analyses showed that vegetative heterokaryon expression is largely additive, but with important variation. Specifically, when investigating the extent of additive and non-additive (dominant and transgressive) allele-specific expression in *N. tetrasperma* heterokaryons, we verified additivity for most genes differentially expressed between homokaryons and heterokaryons in L1 and L6. However, there was substantial variation in L10 (table 1). In this context, it is important to note that L10 showed the greatest divergence, and this is the result of introgression from a closely related species [25]. In this lineage, we have also observed selfish nuclei, which benefit from greater replication and transmission than sister nuclei, at some cost to the heterokaryon [14]. Whether this characteristic of heterokaryosis in L10 is the reason that it differs so substantially from the other lineages in gene expression is not known. However, phenotypic dominance and additive effects have been shown to vary substantially across populations and closely related taxa [44]. Our expression data are consistent with these observations and may reflect variation in the degree of cis versus trans regulation of loci across the different lineages studied here (cf. [45]). Further investigation is needed to reveal the regulatory mechanisms between nuclei in fungal heterokaryons.

### (b) Expression in the sexual stage of development is coregulated between nuclei of heterokaryons

Just as for diploid eukaryotes, it is generally assumed that allelic variants of gene copies in heterokaryons are expressed at levels comparable to their relative dose [16]. However, in mammals, monoallelic expression can result from evolutionary processes such as genomic imprinting [46,47]. In addition, an imbalance in gene dose can be counteracted at the transcriptional level through dosage compensation mechanisms, as has been observed in many species with diverged sex chromosomes [48]. Previous work on six *N. tetrasperma* genes has found some evidence for tissue-specific co-regulation between nuclei to obtain a specific bias in expression [13].

Here, we analysed patterns of allele-specific expression for hundreds of genes in heterokaryons of varying nuclear ratios and from different *N. tetrasperma* lineages. Our findings reveal that expression correlates with the nuclear ratio in the vegetative stage, which suggests that *mat A* and *mat a* nuclei have a similar gene regulation pattern and genes are largely regulated independently within each nucleus. As a result, the level of nucleus-specific gene expression depends more on the nuclear ratio than on transacting gene regulation at this stage of development (figure 3).

By contrast, in the sexual stage of all three lineages, alleles of the two nuclei are expressed at levels that differ from nuclear ratio, suggestive of expression ratio optimization. In L1 and L6, we observe a strong coregulation of gene expression in the sexual stage, as we find a lower slope in the regression between DNA and RNA ratios of *mat A* nuclei for the majority of genes (figure 3). In other words, expression level is more even than predicted by nuclear content. Nuclei of the two mating types may be required to be expressed at similar levels during sexual maturation, and this could be achieved through dosage compensation in the heterokaryon.

Most genes with a more-even expression ratio (slope less than 1) between the two *mat* nuclei in the sexual compared to the vegetative stage are found on the non-recombining region of the mating-type chromosomes (electronic supplementary material, table S5). Previous studies have shown that in both L1 and L6 recombination is suppressed across almost the entire length of the *mat* chromosomes and there is evidence of elevated sequence divergence between the two *mat* chromosomes in these regions [25]. A comparison of eight *N. tetrasperma* lineages revealed that L6 exhibits the highest sequence divergence between the *mat A* and *mat a* chromosomes, more than twice as high as that seen in L1 [25]. Interestingly, we here observe a reduced *mat* expression ratio in sexual heterokaryons of L6 compared to those of L1 (figure 3), which is consistent with stronger selection for transcriptional dosage compensation in L6 due to elevated rates of sequence divergence between mating-type chromosomes [49]. Non-recombining regions can experience loss of gene activity, resulting in dosage imbalance, and future work should examine the degree of degeneration of the non-recombining region of *mat* chromosomes in order to determine the role of dosage compensation in patterns of coregulation of gene expression between nuclei.

In contrast to our results in L1 and L6, in L10 sexual heterokaryons, we find a slope greater than 1 for RNA versus DNA ratios (figure 3). During the sexual stage in L10, although DNA ratios are strongly biased towards *mat A* (which gives a small range of DNA-ratios in this heterokaryon), RNA expression is *mat a* biased (figure 3). In addition, genes with a higher expression in the sexual stage are significantly enriched for *mat a*-biased genes in L10, but not L1 or L6. As strongly *mat A-*biased heterokaryons are infertile [14], a *mat a*-biased expression in these heterokaryons could ensure fertility when nuclear ratio does not exceed 0.9. It seems thus that we observe some sort of compensation of dosage in L10. We have shown that genes whose expression departs from nuclear ratios are mostly located outside of the mating-type chromosome (i.e. on ‘autosomes’) in L10 (electronic supplementary material, table S5). L10 is one of the few *N. tetrasperma* lineages showing elevated divergence on autosomes, which would again select for dosage compensation.

## 5. Conclusion

Taken together, the results of our study advance our understanding of the molecular and evolutionary interplay between different genotypes within the same individual, and therefore intra-organismal genetic heterogeneity. In fungi, delayed karyogamy after mating is a feature that leads to intra-organismal genetic variation in the form of mating-type heterokaryosis. We have previously shown that in *N. tetrasperma*, selection can act at different levels. Although nuclei can compete in replication and transmission into asexual spores, cooperation between nuclear types is required to complete the life cycle of the heterokaryon [14]. Here, we show that the expression of the heterokaryon is predominantly defined by additive effects of the two nuclear types, hinting at nuclear-level control of expression. However, we also verify stage-specific co-regulation of gene expression, which would indicate heterokaryon-level expression regulation. Together with our previous findings of uneven nuclear ratio and complementary fitness optima of the heterokaryon nuclei, we here find further support for the notion that heterokaryons possess an additional level of adaptive flexibility to a changing environment relative to diploids, whereby the stoichiometry of some loci could be altered merely by changing nuclear ratios [4,50].

## Data Availability

RNA-seq data for the homokaryons was generated by Hosseini *et al*. [34] and is deposited at the NCBI Sequence Read Archive under BioProject PRJNA505304. RNA-seq data for the heterokaryons is deposited under BioProject PRJNA768335. The data are provided in electronic supplementary material [51].

## References

[1] Gill DE, Chao L, Perkins SL, Wolf JB. 1995 Genetic mosaicism in plants and clonal animals. Annu. Rev. Ecol. Syst. **26**, 423-444. (10.1146/annurev.es.26.110195.002231)

[2] Santelices B. 1999 How many kinds of individual are there? Trends Ecol. Evol. **14**, 152-155. (10.1016/S0169-5347(98)01519-5) 10322523

[3] Pineda-Krch M, Lehtila K. 2004 Costs and benefits of genetic heterogeneity within organisms. J. Evol. Biol. **17**, 1167-1177. (10.1111/j.1420-9101.2004.00808.x) 15525396

[4] Jinks JL. 1952 Heterokaryosis: a system of adaptation in wild fungi. Proc. R. Soc. Lond. B **140**, 83-99. (10.1098/rspb.1952.0046) 13003914

[5] Otto SP, Orive ME. 1995 Evolutionary consequences of mutation and selection within an individual. Genetics **141**, 1173-1187. (10.1093/genetics/141.3.1173) 8582622 PMC1206839

[6] Raper JR. 1966 Genetics of sexuality in higher fungi. New York, NY: Ronald Press.

[7] Raju NB, Perkins DD. 1994 Diverse programs of ascus development in pseudohomothallic species of *Neurospora*, *Gelasinospora*, and *Podospora*. Dev. Genet. **15**, 104-118. (10.1002/dvg.1020150111) 8187347

[8] Caten CE, Jinks JL. 1966 Heterokaryosis: its significance in wild homothallic ascomycetes and fungi imperfecti. Trans. Br. Mycol. Soc. **49**, 81-93. (10.1016/S0007-1536(66)80038-4)

[9] King RC, Stansfield WD, Mulligan PK. 2006 A dictionary of genetics. Oxford, UK: Oxford University Press.

[10] Anderson JB, Kohn LM. 2007 Dikaryons, diploids and evolution. Washington, DC: ASM Press.

[11] Pontecorvo G. 1946 Genetic systems based on heterokaryosis. Cold Spring Harb. Symp. Quant. Biol. **11**, 193-201. (10.1101/SQB.1946.011.01.021)

[12] Roper M, Ellison C, Taylor JW, Glass NL. 2011 Nuclear and genome dynamics in multinucleate ascomycete fungi. Curr. Biol. **21**, R786-R793. (10.1016/j.cub.2011.06.042) 21959169 PMC3184236

[13] Samils N, Oliva J, Johannesson H. 2014 Nuclear interactions in a heterokaryon: insight from the model *Neurospora tetrasperma*. Proc. R. Soc. B **281**, 20140084. (10.1098/rspb.2014.0084) PMC4046401 24850920

[14] Meunier C, Hosseini S, Heidari N, Maryush Z, Johannesson H. 2018 Multilevel selection in the filamentous ascomycete *Neurospora tetrasperma*. Am. Nat. **191**, 290-305. (10.1086/695803)

[15] Gehrmann T, Pelkmans JF, Ohm RA, Vos AM, Sonnenberg ASM, Baars JJ, Wösten HA, Reinders MJ, Abeel T. 2018 Nucleus-specific expression in the multinuclear mushroom-forming fungus *Agaricus bisporus* reveals different nuclear regulatory programs. Proc. Natl Acad. Sci. USA **115**, 4429-4434. (10.1073/pnas.1721381115) 29643074 PMC5924919

[16] James TY, Stenlid J, Olson A, Johannesson H. 2008 Evolutionary significance of imbalanced nuclear ratios within heterokaryons of the basidiomycete fungus *Heterobasidion parviporum*. Evolution **62**, 2279-2296. (10.1111/j.1558-5646.2008.00462.x) 18637961

[17] Buller AHR. 1931 Researches on fungi. London, UK: Longmans Green.

[18] Kues U. 2000 Life history and developmental processes in the basidiomycete *Coprinus cinereus*. Microbiol. Mol. Biol. Rev. **64**, 316-353. (10.1128/MMBR.64.2.316-353.2000) 10839819 PMC98996

[19] Liu T, Li H, Ding Y, Qi Y, Gao Y, Song A, Shen J, Qiu L. 2017 Genome-wide gene expression patterns in dikaryon of the basidiomycete fungus *Pleurotus ostreatus*. Braz. J. Microbiol. **48**, 380-390. (10.1016/j.bjm.2016.12.005) 28089161 PMC5470450

[20] Song R, Messing J. 2003 Gene expression of a gene family in maize based on noncollinear haplotypes. Proc. Natl Acad. Sci. USA **100**, 9055-9060. (10.1073/pnas.1032999100) 12853580 PMC166437

[21] Springer NM, Stupar RM. 2007 Allele-specific expression patterns reveal biases and embryo-specific parent-of-origin effects in hybrid maize. Plant Cell **19**, 2391-2402. (10.1105/tpc.107.052258) 17693532 PMC2002603

[22] Bell GD, Kane NC, Rieseberg LH, Adams KL. 2013 RNA-seq analysis of allele-specific expression, hybrid effects, and regulatory divergence in hybrids compared with their parents from natural populations. Genome Biol. Evol. **5**, 1309-1323. (10.1093/gbe/evt072) 23677938 PMC3730339

[23] Merino ST, Nelson MA, Jacobson DJ, Natvig DO. 1996 Pseudohomothallism and evolution of the mating-type chromosome in *Neurospora tetrasperma*. Genetics **143**, 789-799. (10.1093/genetics/143.2.789) 8725227 PMC1207337

[24] Ellison CE et al. 2011 Massive changes in genome architecture accompany the transition to self-fertility in the filamentous fungus *Neurospora tetrasperma*. Genetics **189**, 55-69. (10.1534/genetics.111.130690) 21750257 PMC3176108

[25] Corcoran P, Anderson JL, Jacobson DJ, Sun Y, Ni PX, Lascoux M, Johannesson H. 2016 Introgression maintains the genetic integrity of the mating-type determining chromosome of the fungus *Neurospora tetrasperma*. Genome Res. **26**, 486-498. (10.1101/gr.197244.115) 26893460 PMC4817772

[26] Sun Y, Corcoran P, Menkis A, Whittle CA, Andersson SG, Johannesson H. 2012 Large-scale introgression shapes the evolution of the mating-type chromosomes of the filamentous ascomycete *Neurospora tetrasperma*. PLoS Genet. **8**, e1002820. (10.1371/journal.pgen.1002820) 22844246 PMC3406010

[27] Corcoran P, Jacobson DJ, Bidartondo MI, Hickey PC, Kerekes JF, Taylor JW, Johannesson H. 2012 Quantifying functional heterothallism in the pseudohomothallic ascomycete *Neurospora tetrasperma*. Fungal Biol. **116**, 962-975. (10.1016/j.funbio.2012.06.006) 22954339

[28] Samils N et al. 2013 Sex-linked transcriptional divergence in the hermaphrodite fungus *Neurospora tetrasperma*. Proc. Biol. Sci. **280**, 20130862. (10.1098/rspb.2013.0862) 23782882 PMC3712418

[29] Vogel H. 1956 A convenient growth medium for *Neurospora* (Medium N). Microb. Genet. Bull. **13**, 2-43.

[30] Russo V, Sommer T, Chambers J. 1985 A modified Vogel's medium for crossings, mating-type tests, and the isolation of female-sterile mutants of *Neurospora crassa*. Neurospora Newsl. **32**, 10-11. (10.4148/1941-4765.1569)

[31] Galagan JE et al. 2003 The genome sequence of the filamentous fungus *Neurospora crassa*. Nature **422**, 859-868. (10.1038/nature01554) 12712197

[32] Sun Y, Svedberg J, Hiltunen M, Corcoran P, Johannesson H. 2017 Large-scale suppression of recombination predates genomic rearrangements in *Neurospora tetrasperma*. Nat. Commun. **8**, 1140. (10.1038/s41467-017-01317-6) 29074958 PMC5658415

[33] Kurtz S, Phillippy A, Delcher AL, Smoot M, Shumway M, Antonescu C, Salzberg SL. 2004 Versatile and open software for comparing large genomes. Genome Biol. **5**, R12. (10.1186/gb-2004-5-2-r12) 14759262 PMC395750

[34] Hosseini S, Meunier C, Nguyen D, Reimegard J, Johannesson H. 2020 Comparative analysis of genome-wide DNA methylation in *Neurospora*. Epigenetics **15**, 972-987. (10.1080/15592294.2020.1741758) 32228351 PMC7518705

[35] Li L, Stoeckert CJ Jr, Roos DS. 2003 OrthoMCL: identification of ortholog groups for eukaryotic genomes. Genome Res. **13**, 2178-2189. (10.1101/gr.1224503) 12952885 PMC403725

[36] Bolger AM, Lohse M, Usadel B. 2014 Trimmomatic: a flexible trimmer for Illumina sequence data. Bioinformatics **30**, 2114-2120. (10.1093/bioinformatics/btu170) 24695404 PMC4103590

[37] Dobin A, Davis CA, Schlesinger F, Drenkow J, Zaleski C, Jha S, Batut P, Chaisson M, Gingeras TR. 2013 STAR: ultrafast universal RNA-seq aligner. Bioinformatics **29**, 15-21. (10.1093/bioinformatics/bts635) 23104886 PMC3530905

[38] Liao Y, Smyth GK, Shi W. 2014 featureCounts: an efficient general purpose program for assigning sequence reads to genomic features. Bioinformatics **30**, 923-930. (10.1093/bioinformatics/btt656) 24227677

[39] Robinson MD, McCarthy DJ, Smyth GK. 2010 edgeR: a Bioconductor package for differential expression analysis of digital gene expression data. Bioinformatics **26**, 139-140. (10.1093/bioinformatics/btp616) 19910308 PMC2796818

[40] Love MI, Huber W, Anders S. 2014 Moderated estimation of fold change and dispersion for RNA-seq data with DESeq2. Genome Biol. **15**, 550. (10.1186/s13059-014-0550-8) 25516281 PMC4302049

[41] Auger DL, Gray AD, Ream TS, Kato A, Coe EH, Birchler JA. 2005 Nonadditive gene expression in diploid and triploid hybrids of maize. Genetics **169**, 389-397. (10.1534/genetics.104.032987) 15489529 PMC1448873

[42] Landry CR, Hartl DL, Ranz JM. 2007 Genome clashes in hybrids: insights from gene expression. Heredity **99**, 483-493. (10.1038/sj.hdy.6801045) 17687247

[43] Castanera R, Omarini A, Santoyo F, Perez G, Pisabarro AG, Ramirez L. 2013 Non-additive transcriptional profiles underlie dikaryotic superiority in *Pleurotus ostreatus* laccase activity. PLoS ONE **8**, e73282. (10.1371/journal.pone.0073282) 24039902 PMC3764117

[44] Thompson KA, Urquhart-Cronish M, Whitney KD, Rieseberg LH, Schluter D. 2021 Patterns, predictors, and consequences of dominance in hybrids. Am. Nat. **197**, E72-E88. (10.1086/712603) 33625966

[45] Lemos B, Araripe LO, Fontanillas P, Hartl DL. 2008 Dominance and the evolutionary accumulation of cis- and trans-effects on gene expression. Proc. Natl Acad. Sci. USA **105**, 14 471-14 476. (10.1073/pnas.0805160105) PMC2567206 18791071

[46] Gimelbrant A, Hutchinson JN, Thompson BR, Chess A. 2007 Widespread monoallelic expression on human autosomes. Science **318**, 1136-1140. (10.1126/science.1148910) 18006746

[47] Chuang TJ, Tseng YH, Chen CY, Wang YD. 2017 Assessment of imprinting- and genetic variation-dependent monoallelic expression using reciprocal allele descendants between human family trios. Sci. Rep. **7**, 7038. (10.1038/s41598-017-07514-z) 28765567 PMC5539102

[48] Furman BLS, Metzger DCH, Darolti I, Wright AE, Sandkam BA, Almeida P, Shu JJ, Fraser B. 2020 Sex chromosome evolution: so many exceptions to the rules. Genome Biology and Evolution **12**, 750-763. (10.1093/gbe/evaa081) 32315410 PMC7268786

[49] Ma WJ, Carpentier F, Giraud T, Hood ME. 2020 Differential gene expression between fungal mating types is associated with sequence degeneration. Genome Biology and Evolution **12**, 243-258. (10.1093/gbe/evaa028) 32058544 PMC7150583

[50] Davis RH. 1960 Adaptation in pantothenate-requiring *Neurospora*. 2. Nuclear competition during adaptation. American Journal of Botany **47**, 648-654. (10.1002/j.1537-2197.1960.tb07148.x)

[51] Meunier C, Darolti I, Reimegård J, Mank JE, Johannesson H. 2022 Data from: Nuclear-specific gene expression in heterokaryons of the filamentous ascomycete *Neurospora tetrasperma*. *Figshare*. (10.6084/m9.figshare.c.6124035) PMC9363985 35946150

